# Case Report: Unexpected non-pathogenic autoantibodies without clinical involvement following PD-L1 blockade

**DOI:** 10.3389/fimmu.2025.1678680

**Published:** 2025-11-19

**Authors:** Lifan Zhang, Zhe Jin, Shixuan Wang

**Affiliations:** Department of Respiratory and Critical Care Medicine, Peking University First Hospital, Beijing, China

**Keywords:** immune checkpoint inhibitor, cancer immunotherapy, autoantibody, glomerular basement membrane antibody, small cell lung cancer

## Abstract

Several immune checkpoint inhibitors (ICIs) have improved outcomes in extensive-stage small cell lung cancer (ES-SCLC), but immune-related adverse events (irAEs) remain a concern. Beyond irAEs, ICIs may induce various immune changes requiring further investigation. A 74-year-old man with ES-SCLC received atezolizumab plus chemotherapy and developed aspiration pneumonia due to dysphagia. Concurrently, elevated anti-glomerular basement membrane, antinuclear, double-stranded DNA, myeloperoxidase-antineutrophil cytoplasmic, proteinase 3 antibodies were detected without any clinical involvement. Moderate glucocorticoids were administered, and during a 3-month follow-up, he remained entirely asymptomatic, with persistently high antibody titers. The case report presents specific autoantibodies following PD-L1 blockage and questions their pathogenic potential.

## Introduction

Cancer immunotherapy has sparked a revolution in oncology in the past few decades, as it has improved the dismal prognosis of patients with various early- and late-stage cancers. Immune checkpoint inhibitors (ICIs), as essential components of cancer immunotherapy, exert their antitumor effects by blocking the negative costimulatory signals of T cells, mainly the PD-1/PD-L1 or CTLA-4/CD80/CD86 pathways.

Despite this, immune-related adverse events (irAEs) remain a concern. Severe organ-specific or systemic irAEs can be elusive, life-threatening and often result in treatment discontinuation (1). Several mechanisms may contribute to these effects, such as T and B-cell proliferation and infiltration and the production of inflammatory cytokines/chemokines and autoantibodies (2). Nonetheless, it must be recognized that the alterations in the immune system induced by ICIs could be profound and poorly elucidated. Apart from irAEs, the pathogenic potential of subclinical changes in the immune system submerged beneath the iceberg surface remains uncertain and warrants further investigation. Here, we present the first case of a patient with ES-SCLC who developed multiple unexpected non- pathogenic specific antibodies after treatment with atezolizumab.

## Case presentation

**Figure 1:**
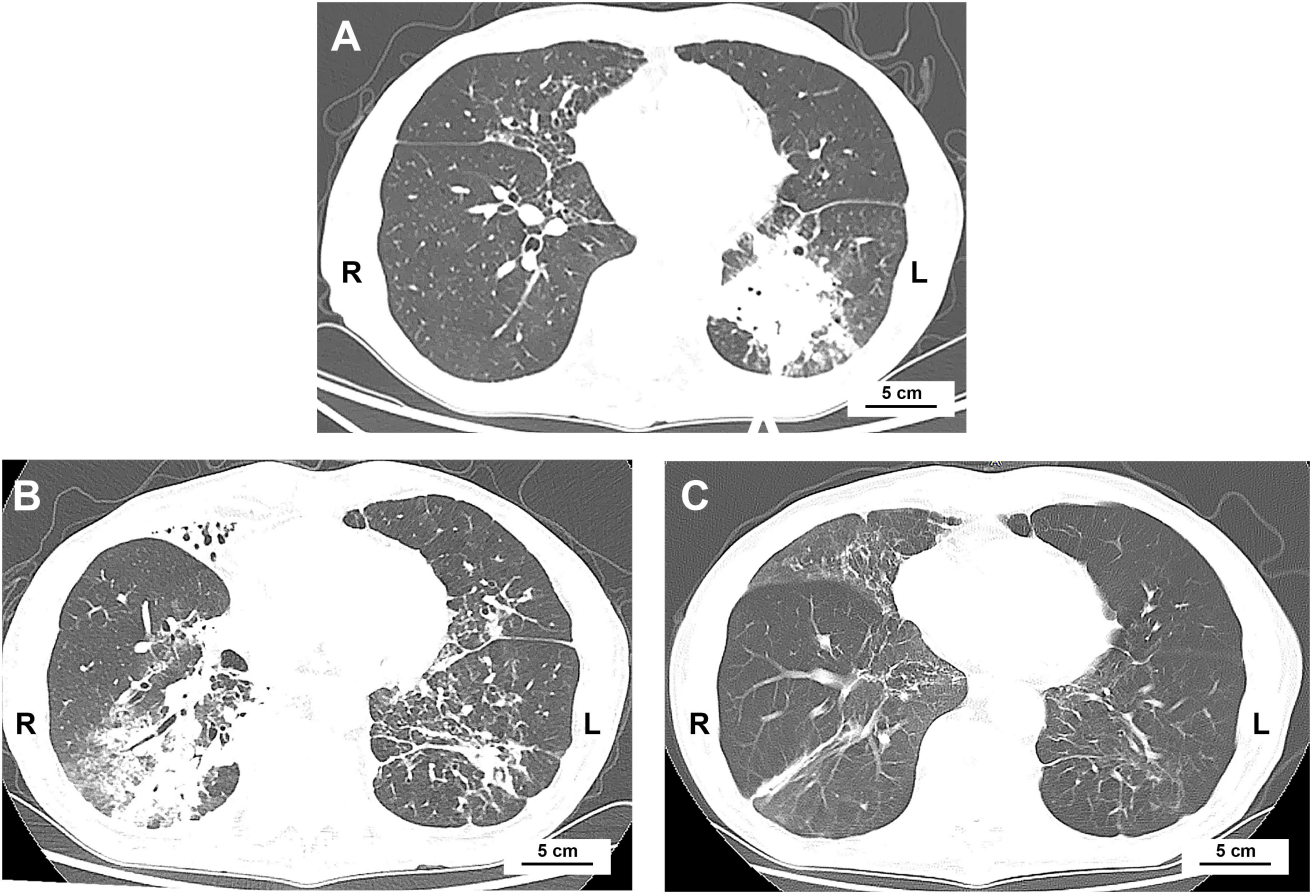
Chest CT scans at baseline and after initiation of atezolizumab. **(A)** Baseline chest CT showing primary lung cancer in the left lower lobe. **(B)** 2 weeks after the second chemotherapy cycle combined with atezolizumab, there was near-complete resolution of the primary lung cancer and new diffuse patchy consolidations in the right middle and lower lobes. **(C)** 4 months after atezolizumab treatment, following anti-infective therapy, patchy consolidations in the right lung had resolved.

**Figure 2:**
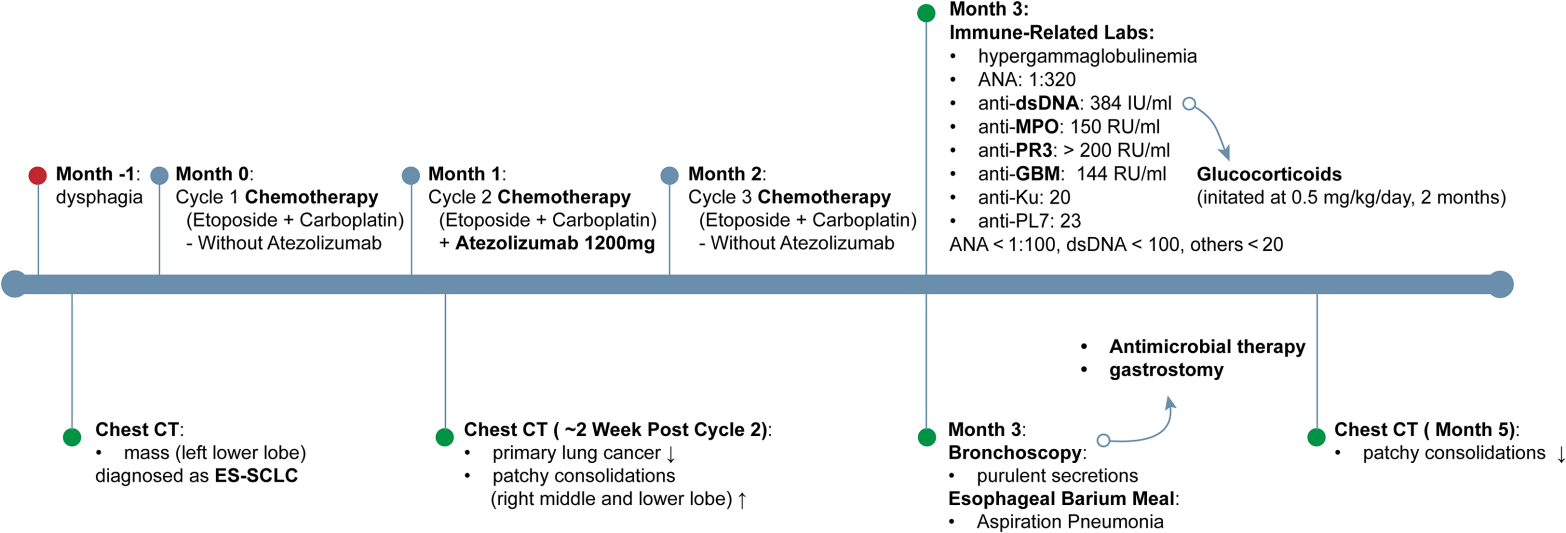
Timeline of clinical course and interventions. Timeline depicting clinical course from one month prior to diagnosis through month 5, outlining symptom onset, imaging changes on chest CT, chemotherapy regimens ± atezolizumab, immune−related laboratory findings, and principal therapeutic interventions. dsDNA, double-stranded DNA; ES-SCLC, extensive-stage small cell lung cancer; GBM, glomerular basement membrane; MPO, myeloperoxidase-antineutrophil cytoplasmic; PR3, proteinase 3.

A 74-year-old man with ES-SCLC was treated with etoposide plus carboplatin, with atezolizumab (1200 mg) added in the second cycle after being withheld in the first due to poor condition. The baseline chest CT scan is shown in **Figure 1A**, and the overall clinical timeline is presented in **Figure 2**. Post-second cycle evaluation showed partial remission with significant reduction of the left hilar lesion and liver metastases; brain imaging revealed resolution of metastases, while chest CT indicated more patchy consolidations in both lungs. After the third chemotherapy cycle, the patient returned with increased bilateral linear consolidations on chest CT (**Figure 1B**), raising concern for ICI-related pneumonitis. He had a 50-year history of smoking five cigarettes daily, prior radiation and chemotherapy for pharyngeal lymphoma, and radiographic features suggestive of ankylosing spondylitis, while without inflammatory back pain or other symptoms of autoimmune diseases.

On admission, laboratory tests showed a white blood cell count of 15,700/mm³ (reference range: 4,000–10,000/mm³; markedly elevated) with neutrophils 86.5% (reference range: 40–75%; elevated), mild anemia, a high-sensitivity C-reactive protein of 140.64 mg/l (reference < 3 mg/l; markedly elevated), and serum creatinine of 1.0 mg/dl (reference range: 0.6–1.2 mg/dl).

Bronchoscopy showed extensive purulent secretions in the trachea and both bronchi, with mucosal swelling and distal narrowing of the left lower lobe bronchus. Neutrophils accounted for 83% of leukocytes in the bronchoalveolar lavage fluid (BALF) from the right B3 bronchus. BALF and esophageal barium meal confirmed aspiration pneumonia with *Klebsiella pneumoniae* infection. After antimicrobial therapy and gastrostomy, the patient’s condition improved, and chest CT showed resolution of the primary patchy consolidations (**Figure 1C**).

Given the initial concern for ICI-related pneumonitis, a series of immune-related laboratory evaluations were performed, and the results were notably confusing. He had hypergammaglobulinemia, with elevated IgG levels of 23.6 g/l (reference range: 7–16 g/l). The antinuclear antibody was positive at a titer of 1:320 (reference < 1:100). The anti-double-stranded DNA (dsDNA) antibody level was markedly elevated at a titer of 384 IU/mL (reference < 100 IU/ml). Antineutrophil cytoplasmic antibody (ANCA) by indirect immunofluorescence (IIF) was negative; however, myeloperoxidase-antineutrophil cytoplasmic (MPO) and proteinase 3 (PR3), measured by ELISA were significantly elevated at 150 RU/ml and > 200 RU/ml, respectively (reference < 20 RU/ml). Moreover, the anti-glomerular basement membrane (GBM) antibody titer was substantially elevated to 144 RU/ml (normal < 20 RU/ml). The anti-Ku and anti-PL-7 antibody titers were 20 and 23, respectively (reference < 20 IU/mL). C3 and C4 were within the normal range.

**Figure 3:**
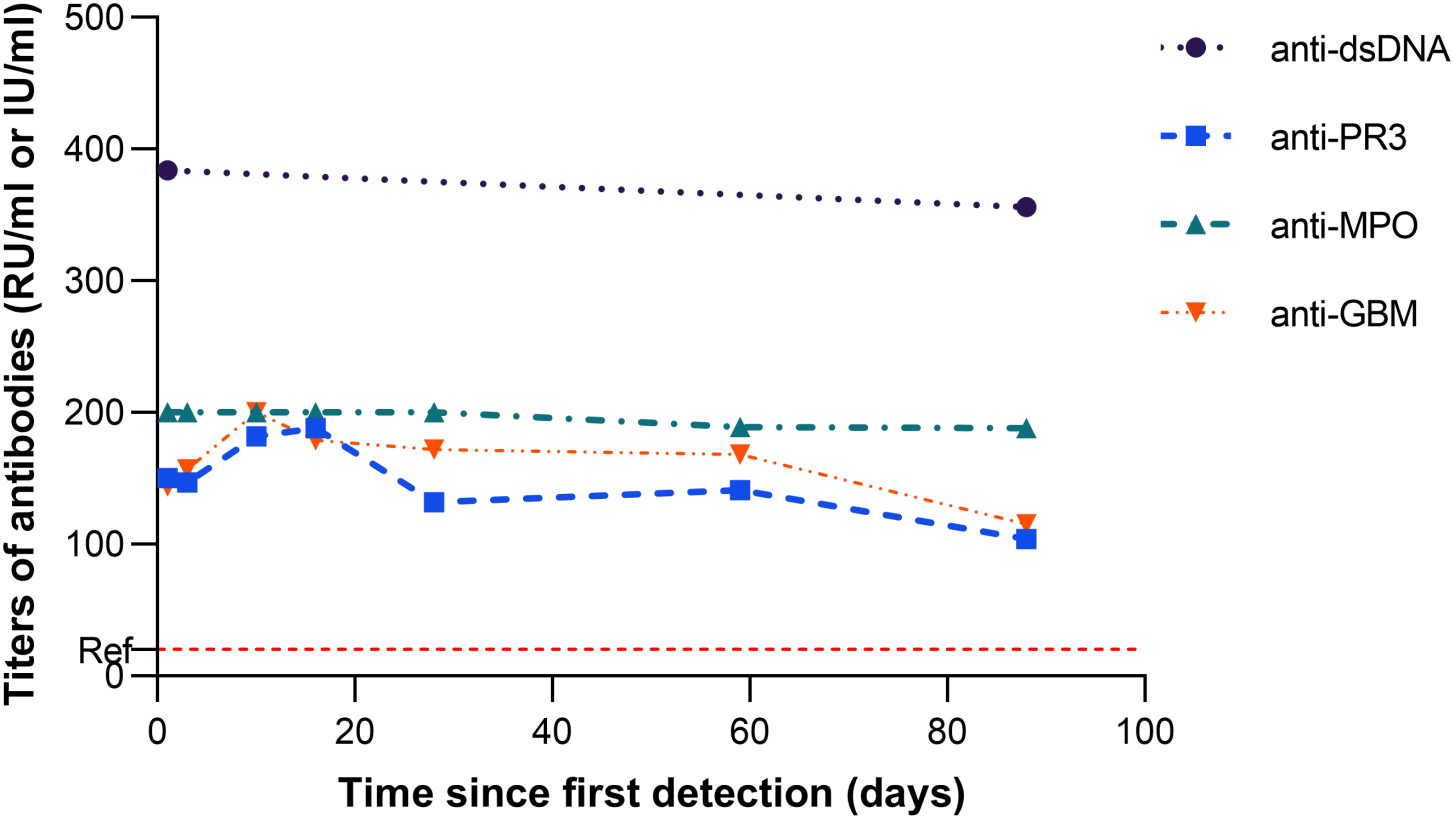
Trajectory of antibody titers (anti−dsDNA, anti−PR3, anti−MPO, and anti−GBM) from their first detection, occurring two months after atezolizumab initiation. The dashed red line marks the reference limit for anti−PR3, anti−MPO, and anti−GBM; the reference limit for anti−dsDNA is 100 IU/ml.

With respect to immunological abnormalities, he exhibited no signs of alopecia, rash, arthralgia, or otolaryngological and neurological involvement. Chest CT and bronchoscopy revealed no typical pulmonary signs of Goodpasture’s syndrome or vasculitis. Urine sediment microscopy showed no clinically significant dysmorphic erythrocytes. Kidney biopsy was advised but declined. In light of concurrent infection and multiple specific antibodies, especially anti-GBM, glucocorticoids (0.5 mg/kg/day) were prescribed. Over the three-month follow-up, no clinical signs of organ involvement were observed. Antibody titers for anti-GBM, PR3, MPO, and dsDNA remained persistently elevated (see **Figure 3**), and glucocorticoids were subsequently tapered and discontinued.

## Discussion

The effects of irAEs differ markedly from those of conventional chemotherapy agents, and their underlying mechanisms remain poorly understood. The case report is the first to present a dramatic autoimmune disorder following cancer immunotherapy characterized by multiple specific autoantibodies, including anti-GBM, PR3, MPO, dsDNA, PL-7 and Ku antibodies, without notable clinical involvement. Given the absence of pre-treatment manifestations of primary autoimmune disease and a favorable response to cancer treatment, it is not attributed to a primary autoimmune disorder or paraneoplastic syndrome, the latter typically parallel to cancer progression (2). The anti-drug immune responses, particularly anti-idiotypic, should be considered. But the detected antibodies were measured using commercial ELISA kits coated with classical autoantigens, without cross-reactivity to anti-drug antibodies. Anti-drug antibody testing should be considered to exclude potential interference.

Autoimmune disorders caused by ICIs could account for the appearance of these antibodies. Although the presence of various specific antibodies is exceedingly rare, these findings reinforce the not strictly antigen-specific mechanism of ICIs. PD-L1 monoclonal antibodies block the coinhibitory pathway activation by targeting PD-L1 on tumor and antigen-presenting cells, thereby enhancing the antitumor response of CD8^+^ T cells. Meanwhile, ICIs can reshape the T-cell repertoire, promoting the differentiation, activation, and proliferation of T cells. The accumulation of highly cytotoxic CD8^+^ T cells, together with a reduction in the frequency of regulatory T cells, contributes to the breakdown of self-tolerance. Alongside pro-inflammatory mediators, these immune shifts facilitate the generation of autoantibodies (2). Posttreatment autoantibody profiles differ by the type of ICIs, likely due to distinct immune-enhancing mechanisms. PD-1/PD-L1 antibodies mainly act in the late phase, modulating CD8^+^ T-cell effector functions, often leaving the autoantibody profile unchanged or reduced. In contrast, CTLA-4 inhibition enhances CD8^+^ CD28^+^ T-cell function, which may lead to more autoantibodies produced by B cells (3). Our patient unexpectedly developed more autoantibodies following PD-L1 antibody treatment. This effect may not be universal, as increased autoantibodies have also been observed in melanoma patients treated with pembrolizumab and nivolumab (4).

The pathogenicity of these specific antibodies induced by cancer immunotherapy remains under scrutiny. Circulating anti-GBM antibodies, whether in primary anti-GBM disease or secondary to immunotherapy, were thought to be highly pathogenic. A few cases have shown that patients receiving ICIs developed high titers of anti-GBM antibodies, all presenting with gross hematuria and progressive renal dysfunction (5–7). Similar to circulating anti-GBM antibodies, dsDNA antibodies are directly associated with disease severity. Dual positivity for PR3 and MPO antibodies is often observed in drug-induced vasculitis; however, no other relevant drugs besides ICIs were administered (8). This dual positivity without immunofluorescent ANCA may also be seen in non-vasculitic conditions such as systemic lupus erythematosus and inflammatory bowel disease (9). High titers of specific antibodies, in the absence of organ involvement, suggest that ICI-induced autoantibodies may partially have distinct pathogenicity from those in *de novo* autoimmune diseases. Most studies on antibody seroconversion following ICI treatment have similarly failed to demonstrate a clear link between specific autoantibodies and organ-specific irAEs (10, 11). A prospective study on autoantibody profiles after CTLA-4 inhibition for melanoma revealed no significant relationship between these antibodies and organ-specific irAEs such as hepatitis and arthritis (10), with similar findings for brain-reactive antibodies and neurological irAEs (11). Autoantibodies following cancer immunotherapy appear to have partial loss of diagnostic and therapeutic relevance. Mechanistically, limited antibody affinity and restricted antigen accessibility may impair antigen–antibody complex formation (12). In addition, insufficient pro-inflammatory or accessory activation signals could hinder the initiation of antibody-dependent or complement-dependent cytotoxicity, processes that require coordinated engagement of Fcγ receptor-mediated or complement pathways together with other immunomodulatory signals.

If similar findings of persistent but non-pathogenic autoantibodies are observed with other PD-L1 inhibitors, or even with other types of cancer immunotherapies, this phenomenon may represent a broader “class effect” rather than a drug-specific response. Beyond the well-recognized enhanced antitumor effects and irAEs, there may also be important subclinical immune alterations, hidden beneath the “iceberg surface,” that deserve equal focus.

With the concurrent presence of multiple antibodies, the patient achieved a favorable treatment response. Autoantibodies may be indirectly associated with therapeutic outcome. Relevant studies have yielded inconsistent conclusions, potentially depending on the autoantibodies involved (4, 10, 13). Antibody seroconversion may indicate activation of the whole immune system. While antibodies targeting autoantigens are produced, the antitumor effect may be enhanced, although the mechanisms may differ.

When the pathogenicity of these autoantibodies remains unconfirmed and concerns about rapid disease progression arise, more caution is needed in selecting optimal strategies. Due to concurrent infection, plasma exchange was initially considered. However, its effect on the antitumor activity of ICIs has not been extensively studied. Although atezolizumab trough levels exceed the threshold for receptor occupancy, repeated plasma exchange could lower PD-L1 antibodies to critically low levels (14, 15). Conversely, short-term glucocorticoids seem to have minimal impact on cancer response (2, 16). Further research is required to provide the evidence needed to opt for observation or immunosuppressive therapy, and reinitiation of immunotherapy.

## Conclusion

Taken together, this case illustrates that cancer immunotherapy can induce multiple nonpathogenic autoantibodies without clinical involvement. Further research is essential to elucidate subclinical immune alterations hidden beneath the “iceberg surface caused by cancer immunotherapy and to refine strategies that harness immune activation to target tumor cells effectively while reducing risks of irAEs.

## Data Availability

The datasets presented in this article are not readily available because of ethical and privacy restrictions. Requests to access the datasets should be directed to the corresponding author/s.
